# Exploring osteosarcopenia from the gut microbiota perspective: mechanistic insights and therapeutic potentials based on the gut-muscle-bone Axis

**DOI:** 10.3389/fmicb.2026.1729870

**Published:** 2026-02-04

**Authors:** Hao-bo Jiang, Jun-qi Zhang, Hao Liang, Li-ying Sun, Chang-qing Deng, Shao-feng Yang

**Affiliations:** 1 Department of Orthopedics, The First Hospital of Hunan University of Chinese Medicine, Changsha, Hunan, China; 2 School of Chinese Medicine, Hunan University of Chinese Medicine, Changsha, Hunan, China; 3 Hunan Provincial Key Laboratory for Prevention and Treatment of Integrated Traditional Chinese and Western Medicine on Cardio-Cerebral Diseases, Hunan University of Chinese Medicine, Changsha, China

**Keywords:** gut microbiota, gut-muscle-bone Axis, musculoskeletal health, osteosarcopenia, therapeutic strategies

## Abstract

The aging society presents a growing challenge in the form of osteosarcopenia (OS). This syndrome is marked by the concomitant deterioration of bone (osteoporosis) and muscle (sarcopenia), and significantly elevates the risks of fractures, disability, and mortality. Despite its clinical relevance, the shared pathophysiology and effective interventions for OS remain elusive. Emerging evidence highlights the gut microbiota (GM) as a critical modulator of musculoskeletal health. This review integrates current evidence to delineate “gut-muscle-bone Axis” framework, summarizing current evidence on how GM dysbiosis may be involved in OS through multifaceted mechanisms, including intestinal barrier disruption, chronic inflammation, endocrine dysregulation, impaired nutrient absorption, and disrupted muscle-bone crosstalk. GM-derived metabolites, such as short-chain fatty acids (SCFAs), interact with immune, metabolic, and hormonal pathways to influence osteoblast/osteoclast activity and muscle protein synthesis. Furthermore, systemic inflammation triggered by GM imbalance exacerbates bone resorption and muscle atrophy. The axis also highlights bidirectional feedback between muscle and bone, mediated by myokines (e.g., irisin) and osteokines (e.g., osteocalcin), which synergistically regulate musculoskeletal homeostasis. Therapeutic strategies targeting GM modulation—such as dietary optimization (plant-based proteins, high-fiber diets), probiotics/prebiotics, exercise, and fecal microbiota transplantation (FMT)—suggest a potential capacity to modulate gut–muscle–bone interactions, which may be relevant to osteosarcopenia-related pathophysiological processes. This review proposes an integrative conceptual framework for understanding the pathogenesis of OS, synthesizing evidence primarily derived from osteoporosis and sarcopenia research, as well as animal and mechanistic studies. While direct clinical evidence in OS remains limited, emerging findings suggest that microbiota-centered strategies may hold potential for future preventive and therapeutic exploration.

## 1 Introduction

With global population aging, the incidence of bone-muscle disorders such as osteoporosis (OP), osteoarthritis (OA), spinal cord injury-related musculoskeletal degeneration (SCI), and osteosarcopenia (OS) is rising (Mohammadi et al., 2023; Salari et al., 2021). Among these conditions, OS — defined as the coexistence of OP and sarcopenia (SP) (Kirk et al., 2020b), resulting in reduced bone mass and diminished muscle mass/strength — deserves particular attention because it combines the risks and functional consequences of both disorders. Epidemiological syntheses and cohort studies report that a substantial proportion of older adults meet criteria for both low bone mass and SP, and OS is consistently associated with higher rates of falls, fractures, frailty, disability and poorer quality of life than either condition alone (Heuberger, 2011; Kirk et al., 2020b; Yu et al., 2014).

Relevant studies have demonstrated that the prevalence of SP is higher among individuals with hip or lower-limb OA (Kemmler et al., 2015). Similarly, individuals with SCI are predisposed to rapid bone loss and secondary SP due to disuse and neuromuscular impairment, which places them at increased risk for osteoporotic fractures and functional decline — conditions that overlap with and can exacerbate OS (Gherle et al., 2024; Winkle et al., 2025). Collectively, these findings indicate that among age-related musculoskeletal disorders, OS is both common and clinically severe. Furthermore, OS elevates the risk of frailty, disability, and mortality in the elderly, and is associated with psychological disorders such as fear, anxiety, and depression (Heuberger, 2011; Kirk et al., 2020b; Yu et al., 2014), posing a significant threat to the health of the aging population. Despite the growing understanding of OS, the shared pathophysiological mechanisms of SP and OP remain elusive, and effective preventive and therapeutic strategies are still limited. This results in a growing burden on both patients’ families and society. Consequently, the effective prevention and treatment of OS have become pressing public health concerns.

In recent years, accumulating evidence suggests that the GM is an important regulator of skeletal health. It modulates the “gut-bone axis” through microbial metabolites, immune regulation, endocrine signaling, and nutrient absorption/biotransformation, profoundly influencing bone metabolism (Chen et al., 2022; Kverka and Stepan, 2024; Lecomte et al., 2023; Li et al., 2023; Merrill and Mangano, 2023; Rettedal et al., 2021; Xiao et al., 2023). Concurrently, growing evidence indicates that specific GM compositions and their functional outputs regulate skeletal muscle mass and function via the “gut-muscle axis.” For instance, the abundance of short-chain fatty acid (SCFA)-producing bacteria such as *Faecalibacterium prausnitzii*, as well as genera like *Lactobacillus* and *Bifidobacterium*, is positively correlated with muscle mass. The underlying molecular mechanisms primarily involve microbial metabolites modulating systemic inflammation, improving insulin sensitivity, promoting protein synthesis, and influencing neuromuscular junction function (Aliwa et al., 2023; Hughes, 2019; O'Brien et al., 2022; Wang et al., 2025; Yu et al., 2024; Zheng et al., 2024; Zhou et al., 2023). Furthermore, a close physiological interaction exists between muscle and bone: muscle contractions not only provide mechanical stimulation to the skeleton but also directly regulate bone formation through complex endocrine mechanisms, such as myokines including Irisin and MYOSTATIN (Herrmann et al., 2020; Hughes et al., 2020; Isaacson and Brotto, 2014). The coordinated regulation of bone and muscle by the GM, as described above, collectively forms the theoretical foundation of the “gut-muscle-bone Axis.”

Building upon the emerging concept of the gut-muscle-bone interrelationship, this review aims to provide a more systematic integration of evidence and intervention strategies specifically focusing on OS as the clinical endpoint. Accordingly, this review explores the pathogenesis of OS through the lens of the GM-mediated “gut-muscle-bone Axis,” aims to integrate current evidence across muscle, bone, and microbiota compartments, and discusses microbiota-targeted strategies as potential and exploratory options that warrant OS-specific validation for this emerging clinical entity.

## 2 Overview of the “gut-muscle-bone Axis”

With the advancement of modern medicine, significant progress has been made in elucidating the interrelationships between the GM, muscle, and bone. However, the precise mechanistic connections among these three components remain incompletely characterized. From a holistic and systemic perspective, this review examines the “gut-muscle-bone Axis” and elucidates its implications for OS, offering new theoretical insights into pathogenesis and potential avenues for targeted therapeutic intervention.

### 2.1 GM and bone

Short-chain fatty acids (SCFAs) are produced in the gut through the fermentation of dietary fiber by beneficial bacteria such as *Bifidobacterium*, *Lactobacillus*, and *Lactococcus*. These SCFAs enhance bone metabolism, increase bone mineral density, and facilitate calcium absorption (Ding et al., 2020; Li et al., 2020). Furthermore, gut-derived metabolites can influence bone metabolism through endocrine pathways by modulating hormones such as leptin, glucagon-like peptide-1 (GLP-1), and casein-derived peptides (Ding et al., 2020; José et al., 2025; Karsenty and Khosla, 2022; Liu H. et al., 2024; Schiellerup et al., 2019). They also regulate bone metabolism by modulating osteoblast (OB) and osteoclast (OC) activities through immune-mediated mechanisms.

Vitamin K synthesized by GM enhances bone mineralization by promoting osteocalcin carboxylation, whereas deficiencies in B vitamins have been strongly associated with reduced bone density (Ding et al., 2020; Li et al., 2020). Moreover, fermentation of dietary fiber by gut bacteria—including *Bifidobacterium*, *Lactobacillus*, and *Lactococcus*—produces SCFAs that are tightly linked to bone metabolism. Animal experiments have demonstrated that SCFAs supplementation significantly increases bone mass in mice, whereas antibiotic-induced SCFAs depletion leads to bone loss. SCFAs such as butyrate and propionate, regulate bone metabolism through two complementary mechanisms: directly enhancing bone density by inhibiting OC differentiation and stimulating OB activity, and indirectly promoting calcium absorption by lowering intestinal pH. Germ-free mouse models exhibit a marked reduction in osteoclast numbers, with bone metabolism restored after transplantation of normal microbiota, confirming that GM maintains bone homeostasis by balancing Th17 and Treg cells. Microbial dysbiosis can lead to excessive Th17 cell proliferation, producing pro-inflammatory cytokines such as IL-17 that activate OCs, whereas Treg cells secrete anti-inflammatory factors such as IL-10 and TGF-β, which inhibit bone resorption (Ding et al., 2020; Karsenty and Khosla, 2022; Liu H. et al., 2024). The gut hormone network plays a multidirectional role in bone metabolism. Gastric inhibitory polypeptide (GIP) promotes bone formation by activating osteoblast receptors, with genetic polymorphisms in its receptor being linked to decreased bone density in humans. GLP-1 regulates bone metabolism bidirectionally via the cAMP/PKA signaling pathway, promoting osteogenic differentiation while inhibiting OC activity. Its receptor agonist, liraglutide, has shown beneficial effects on bone microarchitecture in OP models. Meanwhile, GLP-2 indirectly suppresses bone resorption by modulating parathyroid hormone (PTH) or calcium absorption, whereas peptide YY (PYY) acts via the Y1 receptor to inhibit osteogenesis and enhance bone resorption. Clinical studies indicate that low PYY levels in obese individuals are associated with high bone density, whereas elevated PYY levels following bariatric surgery lead to bone loss (José et al., 2025; Karsenty and Khosla, 2022; Liu H. et al., 2024; Schiellerup et al., 2019).

Overall, GM can enhance bone strength through the synthesis of essential nutrients. Additionally, it can influence bone metabolism either directly via metabolic byproducts or indirectly through immune and hormonal pathways, thereby regulating OB and OC functions.

### 2.2 GM and muscle

The activity, diversity, and metabolic products of the GM are closely associated with muscle mass and function (Li W. et al., 2024; Ticinesi et al., 2019b).

#### 2.2.1 Muscle mass

Research has demonstrated that beneficial microbiota including *Bifidobacterium*, *Ruminococcus*, and *Akkermansia—are positively correlated with muscle mass and strength* (Lahiri et al., 2019; Liu et al., 2021; Mo et al., 2023). This finding highlights the essential role of GM in maintaining muscle morphology and physiological function. Multiple studies consistently indicate that reduced butyrate levels are closely associated with age-related muscle mass loss (Picca et al., 2022). The research by Lv et al. (2021) further confirmed that serum butyrate levels are positively correlated with muscle mass and cross-sectional area, and Mendelian randomization analysis established a positive causal relationship between the capacity of GM to synthesize butyrate and appendicular lean mass. These findings collectively suggest that higher butyrate levels are protectively associated with better muscle mass, while butyrate deficiency may promote the development and progression of muscle loss.

#### 2.2.2 Muscle function

Declines in muscle function adversely affect physical capabilities and independent living (Bean et al., 2003). Beneficial bacteria may influence nutrient biosynthesis and metabolic pathways or activate AMP-activated protein kinase (AMPK) signaling in muscle cells through modulation of bacterial gene expression, thereby regulating muscle metabolism and function (Liu C. et al., 2024; Wang C. et al., 2023). Specific microbial taxa and their metabolites—especially SCFAs such as butyrate, propionate, and acetate—play key roles in enhancing insulin sensitivity and promoting mitochondrial biogenesis, thereby improving energy metabolism and reducing oxidative stress in muscle cells (El-Deeb et al., 2023; Erny et al., 2021; Kumar et al., 2017; Lee et al., 2023).

### 2.3 Muscle and bone

In recent years, numerous studies have demonstrated a strong biomechanical and molecular biological link between bone and skeletal muscle. Bone tissue can sense both external and internal mechanical stresses and transduce mechanical loads into biological signals (Benjamin et al., 2006; Regan et al., 2017b; Schoenau, 2005; Watanabe-Takano et al., 2021; Yin et al., 2024). During physical activity, muscle contraction generates mechanical forces that stimulate bone cells, activating the osteocyte lacuno-canalicular system, thus promoting bone growth (Bergmann et al., 2010; McCrory et al., 2013; Shiba et al., 2015; Vickerton et al., 2014; Zhao et al., 2023). Additionally, skeletal muscle secretes myokines, which enhance OB activity, promote bone tissue metabolism, and help maintain normal bone mineral density (Juffer et al., 2014). Furthermore, bone-derived factors—including osteocalcin, fibroblast growth factor-23 (FGF-23), sclerostin, and prostaglandin E2 (PGE2)—that influence the proliferation and differentiation of myogenic cells, enhance muscle mass, and maintain normal muscle size and strength (Huang et al., 2017; Liu et al., 2017; Mera et al., 2017a; Mera et al., 2017b).

Overall, the relationship between muscles and bone is indirectly maintained through mechanisms such as biomechanics and the secretion of cytokines, which influence the normal growth and metabolism of both muscles and bones (Li et al., 2019).

### 2.4 Gut and muscle-bone

In summary, accumulating evidence suggests that the GM is an important regulatory component of the ‘gut–muscle–bone axis’, interacting bidirectionally with muscle and bone. The dynamic bidirectional communication between muscle and bone further amplifies the impact of GM on the musculoskeletal system. This crosstalk involves biomechanical interactions as well as endocrine and paracrine signaling pathways, in which muscle-derived myokines such as IGF-2 and irisin promote osteogenesis, while bone-derived factors such as osteocalcin and sclerostin regulate muscle metabolism and strength (Mao et al., 2024; Sui et al., 2024). Therefore, the mutual regulation between muscle and bone forms a complex feedback network that integrates mechanical, hormonal, and metabolic cues, thereby reinforcing the systemic influence of GM on both tissues. The detailed mechanisms underlying this bidirectional regulation are discussed in Section 2.3.

Metabolic products of the GM are closely associated with both muscle and bone. For example, a study by Ríos-Covián et al. (2016) reported that SCFAs—primarily acetate, propionate, and butyrate—are generated by fermenting dietary fibers with gut bacteria such as *Bifidobacterium*, *Lactobacillus*, and *Prevotella* species. These SCFAs exert broad systemic regulatory effects. SCFAs enhance intestinal absorption of calcium and magnesium in the gut, thereby stimulating OB activity and increasing bone mineral density (Lucas et al., 2018). Additionally, SCFAs improve insulin sensitivity, enhance muscle glucose uptake (Huang and Czech, 2007), and promote mitochondrial function by activating the AMPK signaling system, which increases muscle metabolism and function (Frampton et al., 2020).

Both muscle and bone are adversely affected by systemic inflammation originating from the gut. Research indicates that gut barrier integrity is compromised in chronic inflammatory conditions such as obesity, metabolic syndrome, age-related inflammation, and acute inflammatory responses. Lipopolysaccharides (LPS), a bacterial byproduct, can also enter the bloodstream and cause systemic inflammation, which harms the musculoskeletal system (Ke et al., 2019). Pro-inflammatory cytokines, including TNF-α and IL-6, stimulate OC differentiation and activity, thereby enhancing bone resorption (Nardone et al., 2021). These inflammatory mediators also promote muscle protein degradation and inhibit protein synthesis, resulting in muscle atrophy and reduced strength. Therefore, systemic inflammation linked to GM dysbiosis may simultaneously impair bone and muscle, a phenomenon commonly observed in elderly individuals and patients with chronic diseases.

The musculoskeletal system is also linked to endocrine regulation within the gut system. Research confirms that several hormones—such as GLP-1, GIP, and leptin—are secreted by gut cells (Baggio and Drucker, 2007). These hormones influence metabolic processes and regulate both bone and muscle through endocrine pathways. GLP-1 and GIP can regulate the activity of OB and OC, promoting bone metabolism balance. Furthermore, leptin demonstrates multifaceted roles in regulating musculoskeletal metabolism. Recent research reveals that leptin enhances local energy expenditure within skeletal muscle. This is achieved through the upregulation of type 2 deiodinase (D2), which boosts the intramuscular conversion of thyroxine (T4) to active triiodothyronine (T3), thereby elevating the basal metabolic rate (Miro et al., 2025). This anabolic energy environment is postulated to be conducive to protein synthesis and tissue repair. Thus, gut-derived hormones regulate systemic energy homeostasis, exerting coordinated effects on both bone and muscle metabolism.

### 2.5 Dominance and feedback in the gut-muscle-bone Axis

The gut–muscle–bone axis is an integrative framework describing interactions among the GM, skeletal muscle, and bone. Within this axis, the GM is increasingly recognized as an important regulatory node that may influence musculoskeletal homeostasis through metabolic, immune, and endocrine pathways. Importantly, regulation is bidirectional: muscle- and bone-derived signals can, in turn, shape gut microbial composition and function. Accordingly, the GM is best viewed as an integral component of a dynamic, multi-organ regulatory network rather than a unidirectional driver. Through metabolite-mediated, immune, and endocrine mechanisms, the GM may modulate both muscle and bone function. Meanwhile, muscle and bone communicate bidirectionally via mechanical loading and the secretion of myokines and osteokines (e.g., osteocalcin), which coordinately contribute to maintaining musculoskeletal homeostasis.

Moreover, the status of muscle and bone can influence the GM indirectly. Following GM modulation, secreted factors from muscle and bone can alter systemic metabolism and provoke inflammatory responses, which in turn may impact GM composition. Thus, the GM, muscle, and bone form a closed-loop feedback system that maintains the balance of the musculoskeletal system and metabolic functions as a whole.

**Figure 1:**
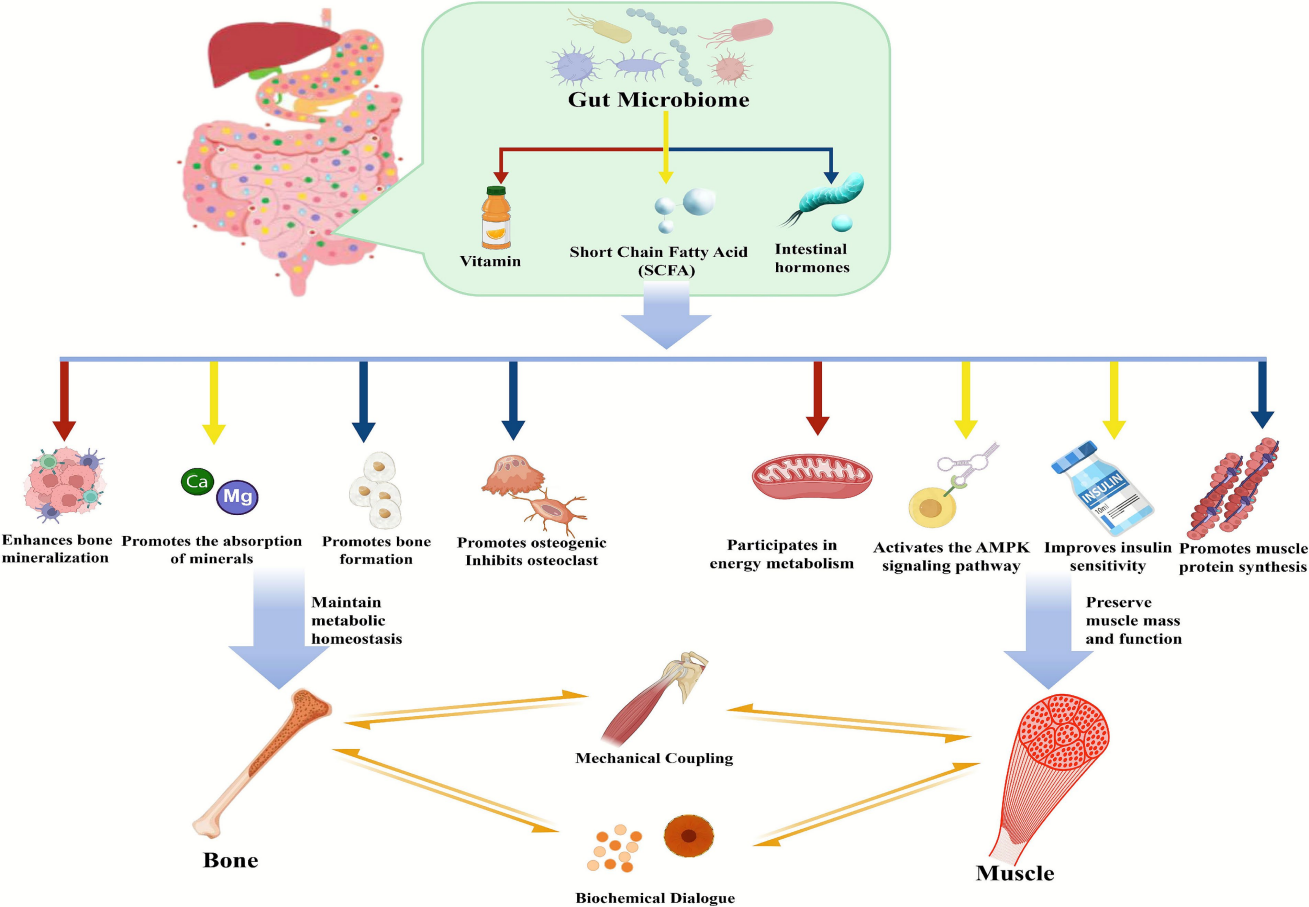
Diagram of the “gut-muscle-bone Axis.”

Accordingly, this article connects these three factors and constructs the “gut-muscle-bone Axis,” as illustrated in Figure 1.

Figure 1 delineates the integral role of the GM in regulating bone and muscle homeostasis, illustrating the pathways through which its disruption may contribute to OS-related changes. The interactions are color-coded.

Vitamin pathways (Red): Gut microbiota-derived vitamins, particularly vitamin K, promote bone mineralization through osteocalcin carboxylation, while B vitamin deficiencies are linked to reduced bone density.

SCFA pathways (Yellow): SCFAS, produced by microbial fermentation of dietary fiber, enhance bone mineral density and calcium absorption, and directly regulate osteoblast and osteoclast activity. Simultaneously, SCFAs activate the AMPK signaling pathway to modulate systemic energy metabolism, improve insulin sensitivity, and help preserve muscle mass and function.

Intestinal hormone pathways (Blue): Gut-derived hormones such as GLP-1 and leptin influence bone metabolism through endocrine signaling and contribute to maintaining muscle metabolic homeostasis.

Additionally, muscle and bone engage in direct crosstalk via mechanical coupling and biochemical dialog, further integrating these three physiological systems.

## 3 Exploring the pathogenesis of OS from the “gut-muscle-bone Axis”

This study aims to explore the pathogenesis of OS by examining interactions within the “gut-muscle-bone Axis.” The following sections explore how GM dysbiosis induces OS through disruption of the intestinal mucosal barrier, immune and endocrine dysregulation, impaired nutrient absorption, and muscle-bone crosstalk, with detailed explanations of each mechanism.

### 3.1 Disruption of the intestinal mucosal barrier

Integrity of the intestinal barrier is essential for maintaining systemic health. The GM preserves this integrity by regulating tight junction proteins in epithelial cells, including occludin, claudin-1, and zonulin. Dysbiosis reduces the expression of these proteins, increasing intestinal permeability. This increased permeability allows endotoxins such as lipopolysaccharide (LPS) to translocate into the bloodstream, triggering systemic inflammation via elevated inflammatory cytokines (e.g., TNF-*α*, IL-6, IL-17) and activating osteoclastogenesis through the RANKL/OPG pathway, thereby triggering systemic inflammation that adversely affects bone and muscle health (Di Vincenzo et al., 2024; Guan et al., 2023). Simultaneously, microbial-derived SCFAs such as acetate, propionate and butyrate—which normally support epithelial barrier integrity and anti-inflammatory signaling—are reduced in dysbiosis, further increasing barrier damage and promoting muscle catabolism through oxidative stress and elevated myokine release (e.g., myostatin) and increased creatinine/cystatin C (Cr/CysC) levels as markers of muscle degradation (Han et al., 2022; Lustgarten, 2019).

Moreover, the disrupted barrier allows dissemination of bacterial metabolites and inflammatory mediators into the circulation, which can impair bone–muscle crosstalk: for instance, altered levels of osteokines (e.g., osteocalcin, sclerostin, FGF-23, PGE2) released from bone may modify muscle regeneration and vice versa, thus linking intestinal barrier dysfunction with the downstream dysregulation of the gut-muscle-bone Axis (Chu et al., 2025; Wang Y. et al., 2023). Together, these mechanisms—increased LPS-driven endotoxaemia, decreased SCFA-mediated protection, elevated inflammatory cytokines, disturbance in Cr/CysC as muscle/bone degradation markers, and altered osteokine signaling—form a pathological cascade initiated by intestinal mucosal barrier disruption which ultimately influences both bone and muscle mass and function.

### 3.2 Immune-inflammatory regulation

The GM dysbiosis perturbs immune homeostasis by modulating the balance between T-helper 17 (Th17) cells and regulatory T cells (Tregs). Specifically, GM imbalance decreases Treg counts and enhances Th17 cell differentiation. The increased Th17 cell population secretes interleukin-17 (IL-17), which promotes OC differentiation and bone resorption, primarily by activating the Receptor Activator of Nuclear Factor kappa-B Ligand (RANKL) pathway. Concurrently, the reduction in Tregs diminishes their secretion of anti-inflammatory factors such as IL-10 and TGF-β, thereby weakening their inherent inhibitory effect on osteoclastogenesis. This collective dysregulation—enhanced pro-osteoclastic signaling via Th17/IL-17 and diminished anti-osteoclastic suppression from Tregs—synergistically exacerbates bone loss (Raphael et al., 2015).

By promoting B cell proliferation, GM dysbiosis raises the RANKL/osteoprotegerin (OPG) ratio and speeds up the creation of OC. On the other hand, GM dysbiosis inhibits Wnt/β-catenin signaling, which lowers OB production of OPG and speeds up bone loss, ultimately leading to OP (Chen et al., 2021).

Studies indicate that a decline in anti-inflammatory bacteria and an increase in pro-inflammatory bacteria contribute to chronic inflammation, markedly impairing muscle mass and function (Farini et al., 2023; Nardone et al., 2021). Inflammatory cytokines have been reported to inhibit insulin-like growth factor 1 (IGF-1) synthesis, thereby decreasing its levels in muscle tissue. In the case of GM dysbiosis, the secretion of pro-inflammatory factors such as IL-6 and TNF-α increases, and these factors inhibit the synthesis of IGF-1, accelerating protein catabolism. These changes suppress muscle protein synthesis, impair muscle metabolism, and ultimately contribute to SP (Yoo et al., 2018).

### 3.3 Endocrine regulation

The GM imbalance disrupts hormone secretion and alters the synthesis of key metabolic products, thereby affecting bone and muscle function.

#### 3.3.1 Hormonal regulation

Flores et al. (2012) reported that GM dysbiosis reduces the abundance of beneficial bacteria, thereby influencing the function of the estrobolome and β-glucuronidase activity, which disrupts estrogen metabolism and enterohepatic circulation, ultimately leading to decreased circulating estrogen levels. This weakens estrogen’s inhibition of OC apoptosis and its regulation of OC proliferation. Moreover, the decrease in estrogen levels enhances OB apoptosis, leading to restricted bone formation and increased bone resorption, ultimately resulting in OP (Zhang L. et al., 2022; Zhang T. et al., 2022; Zhang Y. W. et al., 2022). Additionally, hormones such as gut insulin, thyroid hormone, and PTH are also regulated by GM, thereby affecting bone metabolism (Knezevic et al., 2020). IGF-1 is a key endocrine hormone that plays an important role in cell proliferation and differentiation. GM imbalance disrupts the IGF-1/IGF-1R signaling axis, weakening IGF-1 receptor (IGF-1R) binding to IGF-1. This inhibits OB proliferation, slows bone formation (Hou et al., 2015), ultimately leads to OP.

Research by Saitoh et al. (2017) indicates that growth hormone and IGF-1 are the primary activators of muscle hypertrophy. IGF-1 promotes protein synthesis and inhibits protein degradation through the Akt/mTOR signaling pathway. GM disruption suppresses IGF-1 synthesis, slows protein synthesis, and reduces muscle growth. Hansen (2018) found that estrogen can maintain skeletal muscle protein content by increasing the muscle’s sensitivity to anabolic stimuli, thereby enhancing muscle mass and strength.

Importantly, the hypothalamic–pituitary–adrenal (HPA) axis serves as a central neuroendocrine pathway linking stress, GM, and musculoskeletal health, and therefore must be considered in hormonal regulation. The composition of the GM influences the responsiveness of the HPA axis to stress, as microbial metabolites can modulate HPA output and central stress circuits. Dysregulation of HPA activity leads to chronic elevation of glucocorticoid levels (Rusch et al., 2023), which exert potent catabolic effects: they promote bone resorption while inhibiting bone formation, and induce muscle atrophy by activating proteolytic pathways and suppressing protein synthesis (Hardy et al., 2018; Permpoon et al., 2025). Consequently, the development of OP and SP is made worse by endocrine abnormalities brought on by GM imbalance, which have a direct impact on bone and muscle metabolism.

#### 3.3.2 Metabolite regulation

SCFAs, as key metabolites of GM, exert a bone-protective effect by regulating bone metabolism: they not only support OB function, but also strongly inhibit OC differentiation and bone resorption (Lucas et al., 2018; Srivastava et al., 2022; Yoo et al., 2018). Mechanistically, the two main SCFA subtypes—propionate (C3) and butyrate (C4)—play a dominant role in inhibiting OC activity. They induce early metabolic reprogramming of osteoclast precursor cells (OPCs): by enhancing glycolysis at the expense of oxidative phosphorylation, C3 and C4 trigger cellular energy stress, which in turn downregulates the expression of essential OC-specific signaling molecules, including TNF Receptor-Associated Factor 6 (TRAF6) and Nuclear Factor of Activated T-cells, Cytoplasmic 1 (NFATc1) (Lucas et al., 2018). This regulatory pathway directly blocks RANKL-induced OPC differentiation into functional OCs, thereby suppressing excessive bone resorption.

In contrast, GM imbalance disrupts endogenous SCFA synthesis—particularly the reduction in butyrate production—which weakens the protective effects of SCFAs on bone. A decrease in butyrate levels not only impairs its ability to inhibit OC differentiation but also suppresses OB formation and reduces bone formation rate, ultimately leading to decreased bone mineral density (Jafarnejad et al., 2017; Srivastava et al., 2022). Notably, low circulating SCFA levels have been associated with the progression of OP, as the loss of SCFA-mediated OC inhibition and OB support disrupts the balance of bone remodeling (Lucas et al., 2018; Srivastava et al., 2022).

The SCFAs also alleviate muscle atrophy by upregulating neuromuscular junction proteins such as Rapsyn and low-density lipoprotein receptor-related protein 4 (LRP4) (Qi et al., 2021). Specifically, butyrate promotes mitochondrial biogenesis, enhances skeletal muscle protein synthesis, reduces oxidative stress, and inhibits myocyte apoptosis by downregulating histone deacetylase 3 (HDAC3) and Forkhead box O (FOXO) transcription factors, thereby increasing muscle mass (Walsh et al., 2015).

Therefore, the disruption of SCFAs and other metabolic products secretion caused by GM imbalance, along with endocrine abnormalities, contributes to the regulation of bone formation and resorption, muscle generation and mass, thereby promoting the development of OS.

### 3.4 Nutrient synthesis and absorption

The GM influences bone and muscle metabolism both directly and indirectly by modulating nutrient and energy absorption.

The GM ferments prebiotic fibers into SCFAs, which modify the gut environment and subsequently impact bone health. SCFAs facilitate calcium absorption by reducing intestinal pH and forming mineral complexes (e.g., calcium phosphate), thereby promoting bone growth and maintenance (Whisner et al., 2016). Vitamin D absorption is closely related to bone health. Research has shown that the composition of the GM has a significant impact on vitamin D absorption. Higher Prevotella abundance in the GM is associated with enhanced vitamin D absorption, whereas increased *Bifidobacterium* levels correlate negatively with 25-hydroxyvitamin D concentrations (Boughanem et al., 2023). These findings suggest that specific GM compositions can indirectly influence bone health by modulating vitamin D absorption.

In addition, dietary carbohydrate metabolism is influenced by GM: undigested carbohydrates reach the colon where microbes ferment them, producing SCFAs and other metabolites which feed into host energy metabolism and lipid synthesis pathways. Altered GM composition can shift carbohydrate to SCFA conversion and thereby modulate host glycaemic control, insulin sensitivity and substrate availability for muscle anabolism and bone remodeling (Perler et al., 2023).

In terms of lipid metabolism, the GM modulates bile acid metabolism through bile salt hydrolase activity, thereby influencing lipid digestion and absorption, as well as the bioavailability of fat-soluble vitamins such as vitamin D. Secondary bile acids function as signaling molecules that activate FXR and TGR5 receptors, participating in the regulation of systemic energy homeostasis and inflammatory responses. Dysbiosis may lead to the translocation of endotoxins like LPS, which activates macrophages via the TLR4 pathway, prompting the production of pro-inflammatory cytokines such as TNF-*α*. This, in turn, promotes muscle protein breakdown and bone resorption (Brown et al., 2023).

The GM also plays an important role in regulating protein metabolism. Both dietary and endogenous proteins are hydrolyzed into amino acids and peptides by host and bacterial proteases and peptidases. The resulting amino acids are utilized by host cells and also support microbial growth (Ticinesi et al., 2019a). Gut symbiotic bacteria are crucial for the degradation, synthesis, and absorption of amino acids, especially in the metabolism of key amino acids such as alanine, aspartic acid, glutamic acid, glycine, and tryptophan (Grenier et al., 2023; Oh et al., 2021). Compared with specific pathogen–free mice, germ-free mice show distinct alterations in amino acid distribution throughout the gastrointestinal tract, indicating that the GM is critical for maintaining amino acid balance and host metabolic health (Sasabe et al., 2016). Additionally, the GM can use inorganic nitrogen (such as ammonium salts) to synthesize essential amino acids, and some bacterial strains can even use elemental nitrogen to synthesize organic nitrogen (Cosio and Powers, 2023). The GM is also capable of synthesizing amino acids such as tryptophan, a key substrate for skeletal muscle protein synthesis and metabolism (Jiang et al., 2022). Tryptophan activates the IGF1/p70S6K/mTOR signaling pathway, upregulating genes involved in myofibril synthesis and promoting skeletal muscle growth and function (Dukes et al., 2015).

The GM not only participates in amino acid synthesis but also plays a key role in the synthesis of vitamins. A healthy GM, particularly *Bifidobacterium* and *Lactobacillus*, synthesizes various B vitamins and vitamin K, essential for bone and muscle health (Li et al., 2016; Wong et al., 2018). Compounds produced or modified by the GM, such as folate, vitamin B12, and tryptophan, can enter the systemic circulation and subsequently influence bone and muscle metabolic functions. SCFAs, metabolic products of GM, have been shown to enhance muscle cell growth and development, thereby increasing muscle mass. For instance, propionate—a major SCFA—stimulates glucose uptake in C2C12 myotubes and promotes muscle growth (Han et al., 2014). Thus, SCFAs support normal muscle metabolism and quality.

### 3.5 Muscle-bone crosstalk

Muscle-bone interactions are crucial in the pathogenesis of OS. Disruption of this balance triggers crosstalk, leading to OP and muscle atrophy, which further accelerates OS progression.

#### 3.5.1 Biomechanics between bone and muscle

In exercise-responsive organs, mechanical stimulation directly influences skeletal muscle and bone, particularly during resistance training. Exercise enhances bone mineral density and promotes muscle hypertrophy (Heinonen et al., 1993; O'Bryan et al., 2022; Stokes et al., 2018). Resistance exercise induces skeletal muscle to secrete IGF-1 and promotes the release of osteogenic factors and bone formation. Moreover, elevated IGF-1 contributes to muscle fiber hypertrophy, enhances muscle strength, and prevents muscle loss (Hamrick, 2011; Rojas et al., 2010). Stronger muscles enhance bone loading and stress, whereas weaker muscles decrease loading. When bone strength exceeds muscle strength, bone loss results. Muscle strength determines bone strength (Li et al., 2019). Anatomically, muscle attaches directly to bone; therefore, bone damage removes the muscle’s attachment point, impairing mobility and causing disuse atrophy.

#### 3.5.2 Bone-derived factors

Osteocalcin (OCN): OCN is the most abundant non-collagenous protein in the bone extracellular matrix, and its biological functions in bone and other tissues have been extensively investigated. OCN enhances muscle function by improving insulin sensitivity, energy metabolism, and protein synthesis (Lee et al., 2007; Mera et al., 2017a; Mera et al., 2016).

Sclerostin: Sclerostin is a circulating factor involved in inflammation, insulin resistance, and metabolic regulation. It is produced by osteocytes in bone and acts as an inhibitor of the Wnt/β-catenin signaling pathway (Collette et al., 2010). A Korean study reported that elderly individuals with higher serum sclerostin levels exhibited a lower risk of SP, along with reduced muscle strength and mass (Ahn et al., 2022). Recent evidence indicates that skeletal muscle can also secretes sclerostin. *In vitro* studies using C2C12 myogenic and 2 T3 osteoblastic cells have shown that sclerostin inhibits osteogenic differentiation induced by myogenic medium. Overexpression of sclerostin in skeletal muscle via electroporation reduced bone mass and the bone volume-to-total volume (BV/TV) ratio. These findings suggest that skeletal muscle may represent a novel source of sclerostin, which could act synergistically with bone-derived sclerostin (Magarò et al., 2021). Additionally, studies on circulating sclerostin in skeletal muscle insulin signaling have shown that obese patients exhibit increased sclerostin mRNA expression in cardiac muscle cells. High-fat diet-fed mice also displayed increased skeletal muscle sclerostin expression, whereas *in vivo* inhibition of sclerostin improved insulin sensitivity (Oh et al., 2022). These findings indicate that sclerostin may serve as a key regulator of skeletal muscle metabolism.

Fibroblast Growth Factor 23 (FGF-23): Secreted by OBs and osteocytes, FGF-23 is the first identified bone-derived hormone and is essential for systemic phosphate and vitamin D regulation (Quarles, 2012). Some human phosphate metabolism diseases lead to altered FGF-23 levels, such as X-linked hypophosphatemia caused by PHEX gene mutations (Econs et al., 1998), while Dmp1 mutations have been shown to be the cause of autosomal recessive hypophosphatemic rickets. Interestingly, beyond phosphate metabolism, FGF-23 deficiency induces hypoglycemia and enhances insulin sensitivity in mice (Hesse et al., 2007). Moreover, in Dmp1 mice, a model of hypophosphatemic rickets, force production in the extensor digitorum longus and soleus muscles was significantly reduced, whereas cardiac muscle function remained unaffected (Regan et al., 2017a; Wacker et al., 2016). Faul et al. (2011) demonstrated that FGF-23 induces left ventricular hypertrophy via the calcium/calmodulin-dependent phosphatase/NFAT signaling pathway, indicating its paracrine effect on cardiac muscle cells. Collectively, these studies indicate that bone-derived FGF-23 not only regulates phosphate metabolism but also modulates muscle function.

In addition to the three major bone-derived factors mentioned above, there are also factors such as PGE2, Wnt-3a, and TGF-β. According to the research by Mo et al. (2015) PGE2 can promote the accelerated proliferation and differentiation of myogenic cells. Other studies have confirmed that Wnt-3a enhances the differentiation of C2C12 myogenic cells (Huang et al., 2017). Aberrant TGF-β signaling in bone reduces Ca^2+^-induced muscle contractility, leading to muscle weakness (Waning et al., 2015).

#### 3.5.3 Muscle-derived factors

Skeletal muscle influences bone metabolism through the secretion of myostatin, irisin, and IL-6. Muscle injury increases myostatin secretion, suppressing bone formation and enhancing osteoclastogenesis (Dankbar et al., 2015; Park et al., 2006).

Irisin is a cleaved and secreted fragment of the membrane protein fibronectin type III domain-containing protein 5 (FNDC5) and has been one of the most discussed myokines in the past decade. Irisin, a cleavage product of FNDC5, is actively secreted by skeletal muscle following exercise. In addition to its effects on muscle, it also stimulates the “browning response” in white adipose tissue (Lee et al., 2014). Studies indicate that high-dose irisin upregulates thermogenic genes (e.g., UCP1), whereas low-dose irisin enhances cortical bone density (Boström et al., 2012). Irisin promotes osteogenic gene expression (e.g., OPN and SOST) and enhances OB differentiation and activity *in vitro* (Colaianni et al., 2014). Recent studies demonstrated that FNDC5 knockout mice exhibit significant resistance to ovariectomy (OVX)–induced bone loss compared with sham-operated controls (Kim et al., 2018).

IL-6, when bound to its soluble receptor gp130, can stimulate bone resorption. Mechanically loaded myotubes promote OC formation by secreting IL-6 *in vitro* (Juffer et al., 2014). Additionally, IL-6 increases the differentiation of early OB, and its absence leads to decreased bone mass in mice (Hiscock et al., 2004). Thus, IL-6 may exert dual effects on bone metabolism by stimulating both bone formation and resorption. Beyond the aforementioned muscle-derived factors, IGF-1 and FGF-2 also influence bone metabolism. Several studies have confirmed that IGF-1 and FGF-2 can stimulate bone formation (Hamrick et al., 2010; Kirk et al., 2020a).

**Figure 2:**
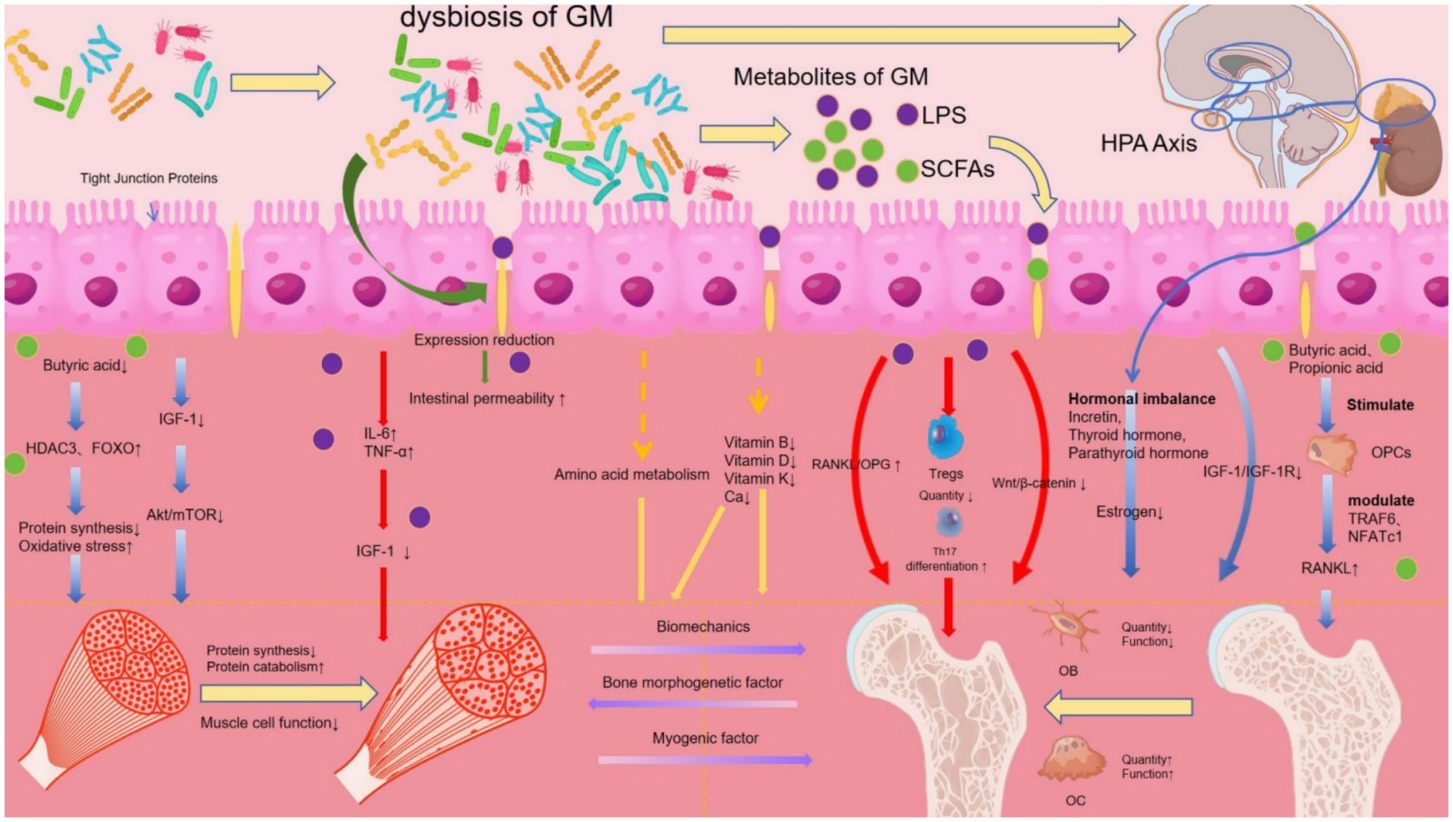
“GM-muscle-bone Axis” perspective on the pathogenesis of OS diagram.

In summary, from the perspective of the “gut-muscle-bone Axis,” the pathogenesis of OS is multifaceted and involves multiple pathways, as shown in Figure 2.

Figure 2 illustrates the pathogenesis of OS through the “gut-muscle-bone Axis” under conditions of GM dysbiosis. The mechanisms are categorized and color-coded.

Inflammatory And Immune Pathways (Red): GM dysbiosis compromises the intestinal barrier, leading to LPS translocation and systemic inflammation (elevated IL-6, TNF-a). This promotes Th17 cell differentiation, upregulates the RANKL/OPG ratio, activates osteoclastogenesis via TRAF6/ NFATc1, and impairs myocyte function through oxidative stress.

Metabolite-Related Pathways (Yellow): Reduced levels of SCFAs like butyrate weaken IGF-1 signaling and increase HDAC3/FOXO activity, resulting in impaired protein synthesis and muscle dysfunction. Declining SCFAs also directly affect bone metabolism.

Vitamin And Hormonal Pathways (Blue): Deficiencies in vitamins B, D, and K, along with hormonal imbalances (e.g., decreased estrogen, incretin, and IGF-1/IGF-1R signaling), lead to reduced bone formation, decreased bone quality and function, and impaired muscle quantity and function.

Collectively, these dysregulated pathways under GM dysbiosis result in the deterioration of both bone and muscle tissues, elucidating a systemic mechanism for OP development.

## 4 Potential methods to alleviate OS by regulating GM

**Figure 3:**
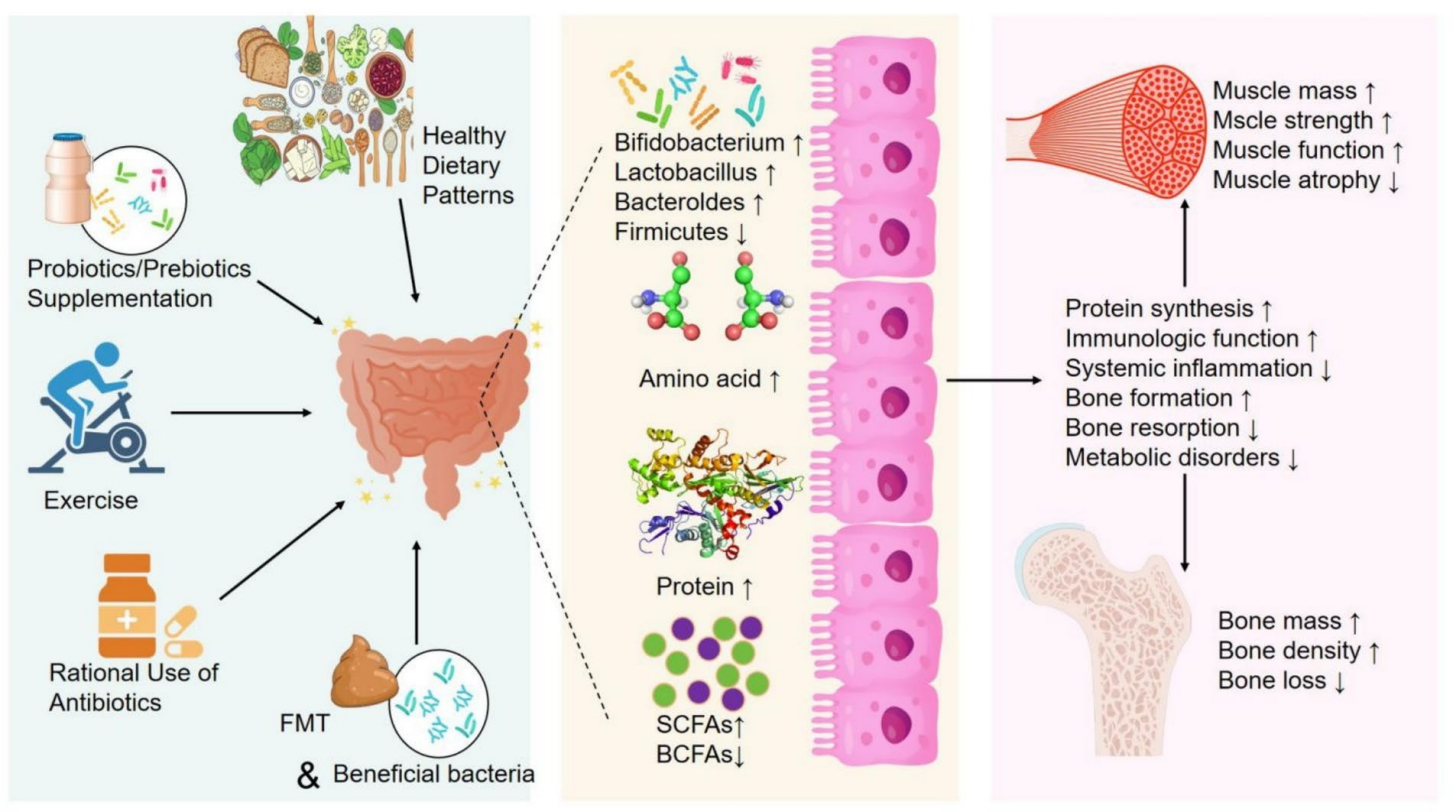
Potential methods to alleviate OS by modulating GM.

The OS is a complex multifactorial condition characterized by the concurrent loss of bone mass and muscle mass. Emerging research highlights the pivotal role of the GM in influencing both bone and muscle health. This section therefore explores potential interventions to alleviate OS from the perspectives of diet, probiotic/prebiotic supplementation, exercise, and fecal microbiota transplantation (FMT), as shown in Figure 3.

Figure 3 summarizes five candidate strategies that may help mitigate OS-related deterioration by modulating the GM. Implementation of these strategies increases SCFAs and protein synthesis while reducing systemic inflammation, which enhance muscle health (increasing mass and strength, improving function) and bone health (promoting formation, inhibiting resorption, increasing bone mass), collectively alleviating OS.

### 4.1 Diet

Diet is fundamental for regulating the GM, maintaining bone health, and preserving muscle function (Devignes et al., 2022; Selvaraj et al., 2024; Zhang et al., 2023). High-protein diets (1.2–1.5 g/kg/day) enhance muscle mass and strength, which may delay SP onset and progression based on current evidence (Motiani et al., 2020). Protein sources significantly influence GM composition. Plant-based proteins tend to promote beneficial bacteria such as Bifidobacterium and Lactobacillus, whereas excessive animal protein intake is associated with increases potentially pathogenic taxa, including *Bacteroides, Alistipes, Bilophila,* and *Clostridium perfringens* (Cotillard et al., 2013; David et al., 2014; Prokopidis et al., 2020; Tomova et al., 2019). Compared to animal proteins, soy protein more effectively promotes Bacteroidetes and reduces serum LPS levels (Butteiger et al., 2016; Reichardt et al., 2014). Furthermore, certain plant-derived *Lactobacillus* and *Bifidobacterium* strains have been associated with increased muscle strength in rodents (Chen et al., 2016; Sprong et al., 2010). Excessive protein intake, especially from animal sources, may negatively affect bone health and should be moderated (Hao et al., 2024). Therefore, based on evidence primarily from studies on SP and OP, it is plausible that high-protein diets prioritizing plant-based sources may help counteract OS. However, direct intervention trials in OS cohorts are needed to confirm this synergistic benefit.

Notably, dietary fiber is another key factor. High dietary fiber consumption produces SCFAs, which enhance mucus secretion, increase antimicrobial peptide levels, and improve intestinal barrier function. In contrast, diets high in animal protein and low in fiber produce toxic metabolites, such as branched-chain fatty acids (BCFAs), leading to mucus degradation, reduced antimicrobial peptide levels, and impaired gut barrier function (Makki et al., 2018). Evidence indicates that high-fiber diets can attenuate the adverse effects of metabolic disorders on muscle mass by increasing GM diversity and circulating free fatty acid concentrations (Hevia-Larraín et al., 2021). Similarly, the European OP guidelines emphasize that physical inactivity and diets low in fiber but high in sugar and saturated fat are associated with promote chronic low-grade inflammation and obesity, thereby exacerbating OP (Biver et al., 2019). Given that chronic inflammation and metabolic dysfunction are also implicated in OS pathogenesis, these dietary factors are likely relevant to OS, though direct evidence is limited.

In summary, current evidence primarily from OP and SP research suggests the potential benefits of a diet high in plant protein, high in fiber, low in sugar, and low in saturated fat may be beneficial for the prevention and management of OS, The proposed mechanisms likely involve GM modulation, reduced inflammation, and improved nutrient absorption. However, dedicated dietary intervention studies in OS populations are warranted to establish specific nutritional guidelines. Dietary interventions represent a promising approach for OS management. Future research should focus on the nutritional status of OS individuals and the OS-specific regulatory role of GM in mediating dietary effects on bone and muscle health within the specific context of OS.

### 4.2 Probiotics/prebiotics supplementation

Probiotics and prebiotics have been reported to potentially contribute to protein synthesis and bone metabolism, effectively promoting muscle mass and bone mineral density (Kang et al., 2024; Schepper et al., 2017; Zhang et al., 2024). Probiotics, particularly *Bifidobacterium* and *Lactobacillus,* are considered essential for maintaining gut homeostasis For instance, *Bifidobacterium* supplementation modulates Gram-negative bacterial populations, maintaining homeostatic levels and reducing toxin absorption (de Marco et al., 2021). Supplementation with *Lactobacillus* improves protein and calcium absorption, decreases intestinal pH, and enhances peristalsis and nutrient uptake (Taslim et al., 2023). However, most of these findings derive from studies not specifically designed for OS, and their direct applicability to OS patients remains to be established.

Prebiotics are indigestible dietary fibers that selectively promote the growth and activity of beneficial gut bacteria (Davani-Davari et al., 2019). Prebiotic intake improves gastrointestinal function and muscle mass by modulating SCFAs. A previous clinical study showed that prebiotic supplements composed of inulin and fructooligosaccharides positively impacted healthy microbiota while enhancing hand strength and endurance in SP patients (Daily and Park, 2022). Fermentation of prebiotic fibers by the GM produces SCFAs and other metabolites that exert bone-protective effects. Dietary prebiotics, such as inulin, fructooligosaccharides, and resistant starch, enhance GM diversity and function, thereby reducing bone loss and improving bone health (Hao et al., 2024; Schepper et al., 2017).

Overall, probiotics and prebiotics show preliminary promise as preventive and therapeutic approaches for OS, though this is largely extrapolated from SP and OP research. However, their efficacy and safety in OS populations remain underexplored. Larger-scale, randomized controlled trials specifically designed for OS are needed to validate these interventions and determine optimal strains, doses, and treatment durations.

### 4.3 Exercise

Substantial evidence indicates that both resistance training and endurance training effectively mitigate the progression of OP and SP. The benefits of exercise on bone and muscle are understood to extend beyond direct mechanical stimulation to include systemic modulation through the GM. Exercise acts as a potent modulator of the gut ecosystem (Hawley et al., 2025). Clinical studies have confirmed that exercise significantly reduces the *Firmicutes/Bacteroidetes* ratio, increases the abundance of SCFA-producing bacteria (e.g., *Bacteroidetes*), and enhances overall microbial diversity, thereby potentially promoting SCFA synthesis (Hu et al., 2020; Moreno-Pérez et al., 2018; Sales and Reimer, 2023).

Within the “gut-muscle-bone Axis,” exercise-induced SCFAs—primarily butyrate, propionate, and acetate—are proposed to serve as crucial signaling molecules. In bone metabolism, SCFAs facilitate calcium absorption and bone mineralization by lowering intestinal pH and forming soluble calcium complexes, while concurrently inhibiting osteoclast differentiation and promoting osteoblast activity (Lucas et al., 2018; Ríos-Covián et al., 2016). In skeletal muscle, SCFAs improve insulin sensitivity, activate the AMPK signaling pathway, promote mitochondrial biogenesis, and upregulate the expression of neuromuscular junction proteins, thereby enhancing protein synthesis and reducing degradation (Carey and Montag, 2021; Qi et al., 2021; Walsh et al., 2015).

Furthermore, studies suggest that resistance training can enhance intestinal barrier integrity by reducing zonulin levels and promoting mucin production, thereby decreasing endotoxin translocation and intestinal inflammation, which in turn helps protect the musculoskeletal system from systemic inflammatory damage (Wagner et al., 2024). The exercise-induced increase in beneficial bacteria such as Bacteroidetes also contributes to mitigating the adverse effects of inflammatory mediators like TNF-α and IL-6 (Hu et al., 2020; Varghese et al., 2024).

In summary, while exercise is a well-established intervention for OP and SP, and its benefits are hypothesized to extend to OS through GM-mediated mechanisms. The development of standardized exercise guidelines—regarding timing, intensity, and modalities—tailored to OS patients represents a critical direction for future research and clinical translation.

### 4.4 Antibiotic use

Antibiotic exposure is widely recognized as a factor that alters GM composition and function, and excessive or inappropriate use can produce deleterious consequences for musculoskeletal health. Broad-spectrum antibiotics, especially when used repeatedly, disrupt microbial diversity, reduce populations of short-chain-fat-acid-producing bacteria, and shift the microbial balance toward taxa associated with inflammation and dysmetabolism. For example, animal studies indicate that in mice treated with high-dose broad-spectrum antibiotics, bone mineral density (BMD) was reduced, osteoblastogenesis inhibited and osteoclast activity increased, relative to untreated controls (Zhao et al., 2021). Likewise, antibiotic treatment in piglets has been shown to altered GM composition and gene expression related to muscle fiber type and lipid metabolism in skeletal muscle, suggesting a link from antibiotics, GM perturbation, muscle quality degradation (Yan et al., 2020). These findings derive from animal studies and have not yet been validated in human OS populations.

Given that both bone and muscle depend on a healthy gut-microbiome ecosystem for optimal nutrient absorption, metabolite production, immune regulation, and endocrine signaling, antibiotic-induced microbial dysbiosis may accelerate the progression of OS by undermining these pathways. However, direct evidence linking antibiotic use to OS onset or progression in humans remains limited and largely inferential.

Therefore, while current understanding supports the rational and judicious use of antibiotics in clinical practice, along with the consideration of microbiota-restorative strategies (e.g., probiotics, dietary fiber, and prebiotics) following treatment, it should be acknowledged tha these recommendations are extrapolated from broader musculoskeletal and microbiological research rather than from OS-specific studies. Further observational and interventional research in at-risk and OS-diagnosed human cohorts is needed to clarify the role of antibiotics in OS pathogenesis and to develop evidence-based antimicrobial stewardship guidelines for this population.

### 4.5 FMT

Recent studies indicate that FMT can regulate and restore GM and its associated metabolites, thereby improving SP-related malnutrition, muscle loss, and overall health, including bone metabolism (Biazzo and Deidda, 2022; Kim et al., 2022; Zhang T. et al., 2022; Zhang Y. W. et al., 2022). FMT can enhance GM and its metabolites, promoting skeletal muscle mass recovery, improving muscle function, and mitigating or preventing SP (Xiao et al., 2024). Fielding et al. (2019) demonstrated that germ-free mice receiving fecal bacteria from elderly individuals with better physical function exhibited significantly greater muscle strength than those receiving bacteria from frail older adults.

Additionally, a review suggested that SCFAs, after FMT, may regulate bone metabolism through multiple mechanisms, including reducing inflammation, enhancing intestinal calcium absorption, promoting osteoblast differentiation via Tregs, and directly inhibiting osteoclast differentiation (Zhang Y. W. et al., 2022). Yan et al. (2016) highlighted that SCFAs indirectly preserve bone mass by modulating serum IGF-1 levels. Thus, FMT may increase SCFA levels, regulate bone metabolism via multiple pathways, maintain bone formation-resorption balance, and help prevent OP.

In conclusion, while FMT represents a promising exploratory approach for modulating both bone and skeletal muscle function based on extrapolation from SP and OP studies, it must be emphasized that its efficacy and safety as a clinical strategy for OS mitigation or prevention remain largely unproven. Future research should prioritize well-designed, OS-specific preclinical models and early-phase clinical trials to evaluate the therapeutic potential, optimal protocols, and long-term outcomes of FMT in this population.

Based on the evidence summarized above, we systematically present in Table 1, the primary intervention strategies for modulating the GM to potentially alleviate OS. It should be noted that most current evidence derives from studies on OP or SP individually; direct evidence from OS-specific intervention trials remains limited. Therefore, the strategies listed are largely extrapolated from related musculoskeletal conditions and warrant further validation in targeted OS cohorts.

**Table 1:** Summary of interventions for OS via gut microbiota modulation.

Intervention type	Study design	Key GM changes	Effects on bone/muscle endpoints	Potential risks/limitations	Evidence sources
Diet	Clinical/Animal studies	• Bifidobacterium, Lactobacillus ↑ • Bacteroides, Alistipes ↑ • SCFAs ↑	• Muscle mass/strength ↑ • Bone density ↑ • Intestinal barrier function ↑	• Excessive animal protein may negatively affect bone health • Long-term adherence challenges	Direct evidence from OS studies
Probiotics	Clinical/Animal studies	• Beneficial bacteria (Bifidobacterium, Lactobacillus) ↑ • SCFA production ↑ • Microbial diversity ↑	• Muscle mass ↑ • Bone density ↑ •Protein/calcium absorption ↑	• Optimal strains/doses not established • Long-term efficacy unknown	
Exercise	Clinical studies	• Firmicutes/Bacteroidetes ratio ↓ • SCFA-producing bacteria ↑ • Microbial diversity ↑	• Muscle strength ↑ • Bone density ↑ • Systemic inflammation ↓	• Lack of standardized protocols • Adherence issues in elderly	Extrapolated from OP/SP studies
Antibiotics	Animal studies	• Microbial diversity ↓ • SCFA-producing bacteria ↓ • Inflammatory taxa ↑	• Bone density ↓ • Osteoblastogenesis ↓ • Muscle quality degradation	• Long-term use harmful • May accelerate OS progression • Findings derived from animal studies; not yet validated in human OS populations	Extrapolated from preclinical models
FMT	Animal/Early clinical studies	• GM structure restoration ↑ • SCFA production ↑ • Microbial diversity ↑	• Muscle mass recovery ↑ • Bone metabolism ↑ • Muscle function ↑	• Safety concerns in elderly • Long-term efficacy unknown • Standardization challenges	Extrapolated from OP/SP studies

Table 1 summarizes intervention strategies potentially relevant to OS-related outcomes through GM modulation. The table concisely integrates information on the types of interventions, their respective study designs, evidence level/source, observed changes in GM composition, resultant effects on bone and muscle endpoints, and potential limitations. This structured overview highlights the current inferential nature of the evidence base for targeting the “gut-muscle-bone Axis” and underscores the urgent need for direct, OS-focused research to develop effective management strategies.

## 5 Discussion

### 5.1 The global clinical burden of osteosarcopenia and evidence

SP and OP are both strongly associated with advancing age, with their prevalence showing a significant upward trend worldwide. Osteoporosis was operationally defined by the World Health Organization(WHO) in 1994 as a bone mineral density 2.5 standard deviations or more below the young adult mean, a criterion that remains the global diagnostic reference standard. Meanwhile, Kanis (1994) has operationally defined osteoporosis, which is now widely integrated into clinical practice (Edwards et al., 2015). In contrast, sarcopenia was only formally recognized as a distinct disease entity in the International Classification of Diseases in 2016, and its diagnostic criteria have not yet been fully standardized globally, posing challenges for accurately assessing its disease burden (Gielen et al., 2023). SP is currently defined according to the revised European Working Group on Sarcopenia in Older People (EWGSOP2) consensus, which emphasizes low muscle strength as the primary diagnostic criterion, supported by low muscle quantity and impaired physical performance (Cruz-Jentoft et al., 2019). It should be noted that OS, which describes the co-occurrence of OP and SP, currently lacks a unified diagnostic definition issued by the WHO (Papaioannou and Kennedy, 2015; Sepúlveda-Loyola et al., 2020). Nevertheless, multiple studies confirm that the two conditions frequently coexist. One clinical study indicates that, depending on the diagnostic criteria used, the prevalence of SP ranges from 28.3 to 55.0%, and it is significantly associated with OP (Zhan et al., 2025). These patients face multiple challenges, including a significantly elevated fracture risk, severely impaired physical function, and a decline in quality of life (Cao et al., 2024). From a socioeconomic standpoint, these two diseases and their complications, such as fractures and disability, contribute to enormous direct medical costs and indirect caregiving expenses, placing continuously growing pressure on healthcare systems (Clynes et al., 2021). Meanwhile, evidence indicates that the significant association between OS and mortality risk cannot be overlooked (Westbury et al., 2024). Patients with OS face a further elevated mortality risk due to fractures, complications, and overall deterioration of health status (Yi et al., 2025).

Although the diagnostic framework for OS remains under development (Sepúlveda-Loyola et al., 2020), its health impact in older adults is increasingly recognized/appears substantial, although standardized diagnostic criteria and high-quality OS-specific clinical evidence remain limited. This syndrome not only substantially elevates individual health risks but also continuously strains public health systems through high medical and caregiving costs, underscoring the urgent need to establish standardized diagnostic criteria and implement targeted prevention and intervention strategies.

### 5.2 Current limitations of preclinical models for osteosarcopenia

Despite the substantial clinical burden imposed by OS, elucidating its underlying pathophysiological mechanisms remains a significant scientific challenge. To investigate the complex nature of this syndrome, research utilizing animal models has become an indispensable preclinical tool. Such studies not only provide critical insights into the pathogenesis of OS but also establish essential platforms for evaluating potential therapeutic interventions. However, existing models primarily capture isolated aspects of OS rather than the full complexity of the condition.

Natural aging models can better reproduce age-related musculoskeletal degeneration, but the modeling cycle is too long (Liang et al., 2022), ovariectomy models are mainly suitable for studying estrogen deficiency-related musculoskeletal metabolic abnormalities and cannot simulate OS caused by other etiologies (Beranger et al., 2015); chemically induced models can rapidly establish OS models, but their drug toxicity may interfere with the accuracy of mechanistic studies (Williams-Dautovich et al., 2017); genetically modified models can target specific gene functions, but their single gene defects often fail to reflect the polygenic interactions seen in human OS (Mito et al., 2015).

Furthermore, there is insufficient research on the temporal dynamics of existing models. As a progressive disease, different stages of OS may involve different pathological mechanisms. However, most current models only reflect the pathological state at a single time point, lacking simulation of the entire disease process. Simultaneously, a gap exists between models and clinical reality: clinically, OS patients often have multiple comorbidities and drug treatments, whereas experimental animal models are often established in strictly controlled environments, making it difficult to fully simulate the actual clinical scenario. This highlights the ongoing need for model refinement and the cautious translation of preclinical findings.

### 5.3 The gut microbiota as a key regulator in OS pathogenesis

An increasing body of evidence indicates that the gut microbiota (GM) is closely associated with musculoskeletal health and may be involved in the pathophysiology of disorders such as OP and SP (Chen et al., 2023; Li et al., 2021). Mechanistically, GM dysbiosis may compromise intestinal barrier integrity, facilitating the translocation of endotoxins like LPS and triggering chronic low-grade systemic inflammation. This inflammatory state has been linked to enhanced osteoclastogenesis via RANKL up-regulation in bone and may impair muscle protein synthesis via down-regulation of anabolic mediators like IGF-1 (Indrio and Salatto, 2025; Li T. et al., 2024). Concurrently, GM-derived metabolites, particularly SCFAs, have been shown to modulate endocrine signaling and enhance nutrient bioavailability, thereby supporting both bone formation and muscle maintenance (Hwang et al., 2025). This crosstalk is integrated within a dynamic network involving muscle and bone, mediated by myokines (e.g., irisin) and osteokines (e.g., osteocalcin, OCN), and mechanical stimuli from muscle contractions that promote bone remodeling (Li et al., 2021). The conceptual framework of the “gut–muscle–bone axis” proposed in this review integrates these interactions, thereby suggesting a potential association between dysregulation of this axis and the progression of OS.

### 5.4 Emerging management strategies targeting the gut-muscle-bone Axis

Within this conceptual framework, several microbiota-oriented strategies have been explored for their potential relevance to OS. Dietary interventions enriched in high-quality protein (particularly plant-based) and dietary fiber may promote SCFA production and increase GM diversity, supporting bone and muscle health (Giron et al., 2022; Hughes and Holscher, 2021). Supplementation with probiotics and prebiotics represents a promising approach, with early data indicating benefits for nutrient absorption, inflammatory modulation, and SCFA production, thereby contributing to improved bone and muscle metabolism (Cooney et al., 2020; Giron et al., 2022). Exercise, particularly resistance training, not only directly benefits muscle and bone but also modulates GM composition, further supporting the axis (Li et al., 2021; Ticinesi et al., 2025). Although in early exploratory stages, fecal microbiota transplantation (FMT) represents a novel approach for restoring GM composition, with preliminary evidence suggesting potential to attenuate muscle loss and enhance bone metabolism, though its safety and efficacy in OS require further validation (Hwang et al., 2025; Li et al., 2021).

### 5.5 Limitations, future directions, and conclusions

Despite these advancements, the current evidence base has notable limitations. Most evidence is still derived from studies focusing solely on OP or SP, with research specifically using OS as a predefined endpoint being relatively limited and scattered. Clinically, there is a scarcity of large-scale randomized controlled trials (RCTs) for OS interventions. Methodologically, assessment protocols for GM and dual musculoskeletal endpoints lack standardization. To overcome these challenges, future research must prioritize several key directions: (1) conducting large-scale, longitudinal RCTs with standardized OS diagnostic criteria and dual primary endpoints; (2) elucidating the precise molecular mechanisms by which GM-derived metabolites influence myocyte and osteocyte function; (3) developing more clinically relevant animal models of OS; and (4) exploring personalized intervention strategies based on individual GM profiles, genetics, and OS phenotypes (Li et al., 2025).

In conclusion, OS is a multifactorial condition arising from complex interactions among aging, metabolic regulation, inflammation, and musculoskeletal decline. This review highlights the gut–muscle–bone axis as an integrative framework that synthesizes current mechanistic and associative evidence. While modulation of the GM through dietary, probiotic/prebiotic, exercise, and potentially FMT-based approaches shows promise, these strategies should be regarded as exploratory rather than established therapies. Further OS-specific clinical and translational studies are required before definitive conclusions regarding causality or therapeutic efficacy can be drawn.
